# Supplementary material for: SARS-CoV-2 infection and cardiovascular or pulmonary complications in ambulatory care: A risk assessment based on routine data
Source: PLoS One. 2021 Oct 21;16(10):e0258914. doi: 10.1371/journal.pone.0258914 (PMC8530335; doi:10.1371/journal.pone.0258914)
Supplement: S1 Table — (PDF) [file pone.0258914.s001.pdf]

S1 Table. International classification of diseases 10th revision (ICD-10) diagnoses included in the multivariable analyses.

Predictors	ICD-10 diagnoses
Anxiety disorder	F41
Asthma	J45-J46
Cancer	Chapter C, D00-D09
Cancer	C00-C80, C97
Hematooncological	C81-C96
Carcinoma in situ (CIS)	D00-D09
Chronic kidney disease (CKD)	N17-N19
Chronic obstructive pulmonary disease (COPD)	J42-J44
Coronary heart disease (CHD)	I20-I25
Dementia	F00-F03
Depression	F32-F34
Type 1 diabetes	E10
Type 2 diabetes	E11
Flu	J09-J11
Pneumonia	J13-J18
Hypertension	I10-I15
Immunodeficiency	D80-D90
Liver disease	K70-K77
Obesity	E66
Tobacco consumption	F17
Vitamin D deficiency	E55
**Outcome (cardiovascular or pulmonary complications)**	
Acute respiratory distress syndrome (ARDS)	J80
Angina pectoris	I20
Apnea	R092
Cardiac arrest	I46
Heart attack	I21
Hypoxia	J96
Pulmonary embolism	I26
Stroke	I63-I64
