# Supplementary material for: SARS-CoV-2 infection and cardiovascular or pulmonary complications in ambulatory care: A risk assessment based on routine data
Source: PLoS One. 2021 Oct 21;16(10):e0258914. doi: 10.1371/journal.pone.0258914 (PMC8530335; doi:10.1371/journal.pone.0258914)
Supplement: S2 Table — Multivariable binary regression model adjusted for age, sex, urbanisation, nursing home living and diseases shown. (PDF) [file pone.0258914.s002.pdf]

S2 Table. Odds ratio (OR) and 95% confidence intervals (CI) for the risk of cardiovascular or pulmonary complications. Multivariable binary regression model adjusted for age, sex, urbanisation, nursing home living and diseases shown.

	Complications risk analysis		
	**OR (95% CI)**	**P value**	**P value***
Tobacco consumption	1.56 (1.35-1.81)	p < 0.001	p < 0.001
Obesity	1.13 (1.01-1.27)	0.032	0.612
Diagnosis			
CHD	2.59 (2.31-2.90)	p < 0.001	p < 0.001
Hypertension	1.65 (1.43-1.90)	p < 0.001	p < 0.001
COPD	1.53 (1.36-1.74)	p < 0.001	p < 0.001
Asthma	1.18 (1.03-1.35)	0.015	0.362
Pneumonia	1.53 (1.31-1.77)	p < 0.001	p < 0.001
Flu	1.06 (0.86-1.30)	0.604	1.000
Immunodeficiency	1.26 (0.96-1.64)	0.098	0.945
CKD	1.25 (1.10-1.42)	p < 0.001	0.015
Liver disease	1.00 (0.89-1.12)	0.994	1.000
Type 1 diabetes	0.89 (0.71-1.12)	0.317	1.000
Type 2 diabetes	1.23 (1.08-1.38)	0.001	0.033
Vitamin D deficiency	1.14 (0.99-1.32)	0.067	0.862
Cancer			
Cancer < 1 year	1.27 (1.05-1.54)	0.015	0.368
Cancer 1-5 years	0.81 (0.68-0.97)	0.020	0.442
Hematooncological < 1 year	1.07 (0.62-1.83)	0.816	1.000
Hematooncological 1-5 years	1.18 (0.75-1.86)	0.477	1.000
CIS < 1 year	1.16 (0.83-1.61)	0.378	1.000
CIS 1-5 years	0.97 (0.77-1.24)	0.826	1.000
Dementia	1.01 (0.86-1.19)	0.886	1.000
Depression	1.18 (1.06-1.31)	0.002	0.072
Anxiety disorder	1.01 (0.89-1.16)	0.836	1.000

*P values corrected for multiple testing.

CHD, coronary heart disease; COPD, chronic obstructive pulmonary disese; CKD, chronic kidney disease; CIS, carcinoma in situ.
