# Supplementary material for: SARS-CoV-2 infection and cardiovascular or pulmonary complications in ambulatory care: A risk assessment based on routine data
Source: PLoS One. 2021 Oct 21;16(10):e0258914. doi: 10.1371/journal.pone.0258914 (PMC8530335; doi:10.1371/journal.pone.0258914)
Supplement: S3 Table — (PDF) [file pone.0258914.s003.pdf]

S3 Table. Characteristics of participants in derivation and validation cohorts; n (%).

	Derivation cohort			Validation cohort		
	Total (n = 34 553)	No complications (n = 33 105)	Complications (n = 1448)	Total (n = 11 518)	No comlications (n = 11 062)	Complications (n = 456)
Gender						
Male	13 322 (38.6)	12 618 (38.1)	704 (48.6)	4472 (38.8)	4265 (38.6)	207 (45.4)
Female	21 231 (61.4)	20 487 (61.9)	744 (51.4)	7046 (61.2)	6797 (61.4)	249 (54.6)
Age						
< 21	3573 (10.3)	3565 (10.8)	8 (0.6)	1245 (10.8)	1245 (11.3)	0 (0.0)
21-30	4928 (14.3)	4907 (14.8)	21 (1.5)	1682 (14.6)	1669 (15.1)	13 (2.9)
31-40	5189 (15.0)	5141 (15.5)	48 (3.3)	1735 (15.1)	1730 (15.6)	5 (1.1)
41-50	5967 (17.3)	5843 (17.6)	124 (8.6)	1884 (16.4)	1845 (16.7)	39 (8.6)
51-60	6975 (20.2)	6738 (20.4)	237 (16.4)	2278 (19.8)	2188 (19.8)	90 (19.7)
61-70	3191 (9.2)	2934 (8.9)	257 (17.7)	1074 (9.3)	987 (8.9)	87 (19.1)
71-80	2128 (6.2)	1813 (5.5)	315 (21.8)	734 (6.4)	646 (5.8)	88 (19.3)
80+	2602 (7.5)	2164 (6.5)	438 (30.2)	886 (7.7)	752 (6.8)	134 (29.4)
Residence						
Sparsely populated rural area	7863 (22.8)	7485 (22.6)	378 (26.1)	2682 (23.3)	2573 (23.3)	109 (23.9)
Rural area	9136 (26.4)	8750 (26.4)	386 (26.7)	3067 (26.6)	2937 (26.6)	130 (28.5)
Urban area	9677 (28.0)	9302 (28.1)	375 (25.9)	3162 (27.5)	3047 (27.5)	115 (25.2)
Large city	7877 (22.8)	7568 (22.9)	309 (21.3)	2607 (22.6)	2505 (22.6)	102 (22.4)
Nursing home living	3524 (10.2)	3052 (9.2)	472 (32.6)	1203 (10.4)	1060 (9.6)	143 (31.4)
Tobacco consumption	3295 (9.5)	3044 (9.2)	251 (17.3)	1080 (9.4)	1021 (9.2)	59 (12.9)
Obesity	7099 (20.5)	6600 (19.9)	499 (34.5)	2305 (20.0)	2151 (19.4)	154 (33.8)
Diagnosis						
CHD	3260 (9.4)	2605 (7.9)	655 (45.2)	1075 (9.3)	873 (7.9)	202 (44.3)
Hypertension	12 170 (35.2)	11 009 (33.3)	1161 (80.2)	4053 (35.2)	3693 (33.4)	360 (78.9)
COPD	3658 (10.6)	3214 (9.7)	444 (30.7)	1208 (10.5)	1077 (9.7)	131 (28.7)
Asthma	5210 (15.1)	4931 (14.9)	279 (19.3)	1696 (14.7)	1602 (14.5)	94 (20.6)
Pneumonia	1843 (5.3)	1620 (4.9)	223 (15.4)	621 (5.4)	562 (5.1)	59 (12.9)
Flu	2440 (7.1)	2354 (7.1)	86 (5.9)	833 (7.2)	807 (7.3)	26 (5.7)
Immunodeficiency	1042 (3.0)	988 (3.0)	54 (3.7)	368 (3.2)	351 (3.2)	17 (3.7)
CKD	2934 (8.5)	2452 (7.4)	482 (33.3)	998 (8.7)	849 (7.7)	149 (32.7)
Liver disease	4941 (14.3)	4540 (13.7)	401 (27.7)	1702 (14.8)	1570 (14.2)	132 (28.9)
Type 1 diabetes	715 (2.1)	625 (1.9)	90 (6.2)	221 (1.9)	198 (1.8)	23 (5.0)
Type 2 diabetes	4072 (11.8)	3530 (10.7)	542 (37.4)	1301 (11.3)	1135 (10.3)	166 (36.4)
Vitamin D deficiency	3518 (10.2)	3313 (10.0)	205 (14.2)	1156 (10.0)	1080 (9.8)	76 (16.7)
Cancer	4135 (12.0)	3737 (11.3)	398 (27.5)	1405 (12.2)	1284 (11.6)	121 (26.5)
Dementia	2086 (6.0)	1741 (5.3)	345 (23.8)	676 (5.9)	576 (5.2)	100 (21.9)
Depression	11 168 (32.3)	10 462 (31.6)	706 (48.8)	3510 (30.5)	3300 (29.8)	210 (46.1)
Anxiety disorder	5561 (16.1)	5277 (15.9)	284 (19.6)	1707 (14.8)	1622 (14.7)	85 (18.6)

CHD, coronary heart disease; COPD, chronic obstructive pulmonary disease; CKD, chronic kidney disease.
