# Supplementary material for: SARS-CoV-2 infection and cardiovascular or pulmonary complications in ambulatory care: A risk assessment based on routine data
Source: PLoS One. 2021 Oct 21;16(10):e0258914. doi: 10.1371/journal.pone.0258914 (PMC8530335; doi:10.1371/journal.pone.0258914)
Supplement: Attachment — Submitted filename: Response to Reviewers.docx [file pone.0258914.s004.docx]

**Rebuttal Letter**

Dear Editor, dear Reviewers,

Thank you for reviewing our manuscript and for your helpful comments and suggestions for improvement. In the following, we address these point by point (line references refer to the version of the tracked changes).

To improve readability, we have formatted our responses in italics.

Journal requirements:

When submitting your revision, we need you to address these additional requirements.

1. Please ensure that your manuscript meets PLOS ONE's style requirements, including those for file naming. The PLOS ONE style templates can be found at

<https://journals.plos.org/plosone/s/file?id=wjVg/PLOSOne_formatting_sample_main_body.pdf> and

[https://journals.plos.org/plosone/s/file?id=ba62/PLOSOne_formatting_sample_title_authors_affiliations.pdf](about:blank)

*R: Thank you for the templates. We hope to meet the Journal style requirements by updating the following parts:*

- *In the author names we have moved the commas to the end.*
- *We have now updated figure citations (e.g. „Figure 1“ is now „Fig 1“, titles are bold) and figure files naming (e.g. „Figure 1.tiff“ is now „Fig1.tif“).*
- *We have formatted table citations (titles are now bold).*
- *We have now updated Supporting Information citations in the text and listed Supporting Information captions at the end of the manuscript in a section titled “Supporting information”. In addition, we uploaded Supporting Information files separately.*
- *We have updated the font size for the headings.*

2. Please provide additional details regarding participant consent. In the ethics statement in the Methods and online submission information, please ensure that you have specified (1) whether consent was informed and (2) what type you obtained (for instance, written or verbal, and if verbal, how it was documented and witnessed). If your study included minors, state whether you obtained consent from parents or guardians. If the need for consent was waived by the ethics committee, please include this information.

If you are reporting a retrospective study of medical records or archived samples, please ensure that you have discussed whether all data were fully anonymized before you accessed them and/or whether the IRB or ethics committee waived the requirement for informed consent. If patients provided informed written consent to have data from their medical records used in research, please include this information.

*R: The data are pseudonymized (the data are not technically anonymized because the source data with identifying information are held by the Bavarian Association of Statutory Health Insurance Physicians). We hope to meet your requirements by reformulating ethics statement: „The underlying data for this study are pseudonymized and the study was approved by the Ethics Commission of the Technical University of Munich (Ethikkommission der Technischen Universität München) (approval No 673/20 S-EB).“ In addition, we addressed details regarding participant consent in the Methods section, specifically that no further participant consent was required because the analyses were based on secondary billing data and were conducted in accordance with the German guideline "Good Practice in Secondary Data Analysis." (see lines 79-81)*

3. We note that you have indicated that data from this study are available upon request. PLOS only allows data to be available upon request if there are legal or ethical restrictions on sharing data publicly. For information on unacceptable data access restrictions, please see <http://journals.plos.org/plosone/s/data-availability#loc-unacceptable-data-access-restrictions>.

In your revised cover letter, please address the following prompts:

a) If there are ethical or legal restrictions on sharing a de-identified data set, please explain them in detail (e.g., data contain potentially identifying or sensitive patient information) and who has imposed them (e.g., an ethics committee). Please also provide contact information for a data access committee, ethics committee, or other institutional body to which data requests may be sent.

b) If there are no restrictions, please upload the minimal anonymized data set necessary to replicate your study findings as either Supporting Information files or to a stable, public repository and provide us with the relevant URLs, DOIs, or accession numbers. Please see <http://www.bmj.com/content/340/bmj.c181.long> for guidelines on how to de-identify and prepare clinical data for publication. For a list of acceptable repositories, please see <http://journals.plos.org/plosone/s/data-availability#loc-recommended-repositories>.

We will update your Data Availability statement on your behalf to reflect the information you provide.

*R: We updated Data Availability statement to reflect Journal requirements: „The data are held by the Bavarian Association of Statutory Health Insurance Physicians (BASHIP) and availability is restricted by a contractual agreement. The data are therefore not publically available due to data protection regulations, but may be obtained from the authors upon reasonable request and with the consent of the BASHIP (versorgungsforschung@kvb.de).“*

Reviewers' comments:

Reviewer's Responses to Questions

**Comments to the Author**

*R: Questions without reviewer comments (1.-4. and 6.) and notes have been removed to improve readability.*

5. Review Comments to the Author

**Reviewer #1:** Karapetyan and colleagues initial an interesting study to investigate the factors and building model for COVID-19 and following complications using Germany PCR database. The result showed good accuracy, they provided technical information detailly. However, I have some questions about the study design and the source material.

*R: Thank you for your review and the valuable comments.*

(1) Was authors investigate the source of study populations? For example, some patient felt uncomfortable so they decided to take PCR test by themselves, others could be notice or required by government. In my point of view, there were quite different in the risk of SARS-CoV-2 infection, if this factor is available and meaningful for model building, I would happy to see revised result, and please describe some related regulation briefly to help reader understand Germany’s policy.

*R: Thank you for your note. We also think it would be interesting to include this factor in the models. However, the distinction between codes describing the reason for taking the PCR test did not exist at the beginning of the pandemic; it was introduced during the third quarter in 2020 and, unfortunately, was not used consistently by physicians after introduction, as we have seen in our data, so we could not include this factor in the model. We do now describe Germany’s testing strategy in the methods section (see lines 72-75).*

(2) According to your study setting, residence area was categorized to urbanization level and including in the model. Urbanization was an interesting factor, also, provide some information of people’s probability to working, communication, interaction with other people. But my question is, an area, its local COVID-19 prevalence, maybe more important than the urbanization. Could you consider this factor in your analysis?

*R: Thank you for this valuable point. We indeed did not consider this factor in our analysis. However, our objective was not to follow infection chains or to describe hotspots, but to adjust our analyses to the more general effects of different settlement and health care supply densities. We added this to the methods section and now we write: „To adjust for different settlment and health care supply densities we included a measure of urbanization …“ (see lines 106-107).*

(3) Finally, in Results line 186, there was written “For the analysis of defined complications the cohort of test-positives was reduced to 46 071 participants with available data for the first quarter after the index quarter of PCR test”. What is it mean about “available”? Please address in detail.

*R: Thank you for addressing this. We hope to make this clear by reformulating the sentence (see line 194).*

**Reviewer #2:** In this study, the authors used ambulatory claims data to determine possible risk factors for (1) COVID infection, using a test-negative design (2) cardiovascular or pulmonary complications in patients with positive COVID tests, using a cohort design. The authors also developed a rule to predict the risk of cardiovascular or pulmonary complications among those with positive COVID tests.

Overall, this is a well-designed study that answers important clinical questions using available data. I only have some minor points for the authors to consider:

*R: Thank you for your review and the supportive comments.*

1. In the model mentioned in page 11 (line 208-217), do the P values account for multiple comparison and the usage of stepwise regression? If not, it can be serious inflated and should not be used to argue that the interaction terms are statistically significant (so a separate risk factor model should be constructed for test-positives). Since the authors are essentially testing the relative fit between models with and without interaction terms, maybe it would more sound to calculate the likelihood ratio between the two models and use bootstrap to test for its significance.

*R: Thank you very much for raising this important point. The mentioned P values indeed do not account for multiple comparison, however we choose the model based on Akaike’s Information Criterion (AIC). We now removed the P values and pointed out that we compared the models based on a descriptive likelohood-ratio test. In the methods section we now write: “Goodness-of-fit of these nested models was compared by a descriptive likelihood-ratio test without formal adjustment for AIC-based model selection.” (see lines 140-141) Further, in the results section we write now: “… a multivariable regression model with forward stepwise variable selection by AIC included interaction effects between the PCR test result and the investigated risk factors …” (see lines 218-223). We hope this will clear up any confusion.*

2. In a test-negative design, it is optimal that the symptoms prompting the patients to undergo testing are similar between test-positives and test-negative, so as to prevent certain risk groups showing different patterns of probabilities of receiving tests, which then introduces bias. For example, if obese patients are more alert to anosmia than others because they know they're at higher risk of complications once infected by COVID, then the testing rate of test-positive obese patients would be higher than others, leading to the conclusion of "higher risk of infection" when the test-negative design is applied. The authors may discuss more on possible scenarios that violate the assumptions of test-negative designs.

*R: Thank you for raising this important point. We discussed it in the limitations detailed in the discussion section (see lines 314-317).*

3. In the second paragraph of the discussion section, the author attributed the attenuation or inversion of risk to the behavioral adjustment. In a test-negative design, this argument is only valid if the behavioral adjustment decreases the COVID (those who would be test-positive) infection rate more than common cold (those who would be test-negative). Do behavior adjustments demonstrate differential protective effects against COVID vs common cold?

*R: We agree with the point that the argument would be valid if behavioral adjustment is more successful in reducing risk for COVID-19 than risk for other infections. However, this is only true in a setting where only symptomatic participants are tested. Because we also have asymptomatic participants in our cohort, i.e. within the test-positives and within the test-negatives, we cannot directly state that. We therefore now write: „The latter might be explained with possible behavioural adjustment in the patients belonging to respective vulnerable subgroups, however, this assumption might be impaired as the cohort of tested participants also includes asymptomatic participants.” (see lines 268-269)*

4. Are all patients tested symptomatic, or are there screening tests for asymptomatic patients? If the latter is the case, differential screening rate among different risk groups may also introduce bias.

*R: There have also been PCR tests for asymptomatic patients, e.g. for travelers, asymptomatic individuals in health care or other vulnerable sectors, contacts (with criteria of exposure or disposition) etc. This can indeed introduce bias to the assessment of total risks. However, we assume that the assessment of relative risks is unaffected. Therefore, the association between risk factors and the outcome, the estimated odds ratios, should be unbiased.*

5. The authors mentioned that the recording of negative test results was optional for physicians (line 304). This could introduce bias if the behavior of selective recording differs between patient groups. For example, the physician is more willing to record the negative tests of a smoking patient than a non-smoking patient, smoking would be falsely considered as a protective factor.

*R: Thank you very much for this valuable point. We added it to the limitations detailed in the discussion section (see lines 320-322).*

6. The predictive model had adequate performance in the internal validation. However, the generalizability of the model should be assessed by external validation. The authors may list this as one of the limitations of the study.

*R: We agree with this point and add this now as one of the limitations of our study (see lines 306-307).*

7. In the discussion section line 284, PPV = 13.3% may seem low to readers. Maybe the authors can reiterate the prevelance of complications (~4%) to demonstrate why this PPV is acceptable.

*R: Thank you for your suggestion. We now explain this in the discussion section (see lines 295-297).*

While revising your submission, please upload your figure files to the Preflight Analysis and Conversion Engine (PACE) digital diagnostic tool, <https://pacev2.apexcovantage.com/>. PACE helps ensure that figures meet PLOS requirements. To use PACE, you must first register as a user. Registration is free. Then, login and navigate to the UPLOAD tab, where you will find detailed instructions on how to use the tool. If you encounter any issues or have any questions when using PACE, please email PLOS at [figures@plos.org](mailto:figures@plos.org). Please note that Supporting Information files do not need this step.

*R: We uploaded our figure files to the Preflight Analysis and Conversion Engine (PACE) digital diagnostic tool to ensure that our figures meet PLOS requirements. We have updated our figures accordingly.*
