# Supplementary material for: SARS-CoV-2 infection and cardiovascular or pulmonary complications in ambulatory care: A risk assessment based on routine data
Source: PLoS One. 2021 Oct 21;16(10):e0258914. doi: 10.1371/journal.pone.0258914 (PMC8530335; doi:10.1371/journal.pone.0258914)
Supplement: Attachment — Submitted filename: Response to Reviewers.docx [file pone.0258914.s005.docx]

**Rebuttal Letter**

Dear Editor, dear Reviewers,

Thank you very much for considering our manuscript as potentially acceptable for publication in *PLoS One*. We incorporated all of the comments into the revised version and attached a point by point response to all comments (line references refer to the version of the tracked changes). We would be pleased to see the actual version published in *PLoS One*.

ACADEMIC EDITOR: Please mention and discuss your assumption in the Discussion section (please refer to the reviewer comment).

*R: Thank you for your suggestion. We have discussed our assumptions in the Discussion section as proposed from the Reviewer (see lines 335-339).*

Journal Requirements:

Please review your reference list to ensure that it is complete and correct. If you have cited papers that have been retracted, please include the rationale for doing so in the manuscript text, or remove these references and replace them with relevant current references. Any changes to the reference list should be mentioned in the rebuttal letter that accompanies your revised manuscript. If you need to cite a retracted article, indicate the article’s retracted status in the References list and also include a citation and full reference for the retraction notice.

*R: Thank you for your note. We have checked our reference list. All articles are still available and none have been retracted. We have noted that the article by Ioannisids J. (2020) has now been published in Bulletin of the World Health Organization and have changed the reference accordingly:*

- *Ioannidis JPA. The infection fatality rate of COVID-19 inferred from seroprevalence data. medRxiv. 2020;2020.05.13.20101253 🡪 Ioannidis, J. P. Infection fatality rate of COVID-19 inferred from seroprevalence data. Bull World Health Organ. 2021;99(1), 19.*

Reviewers' comments:

Reviewer's Responses to Questions

**Comments to the Author**

6. Review Comments to the Author

**Reviewer #2:** 4. Are all patients tested symptomatic, or are there screening tests for asymptomatic patients? If the latter is the case, differential screening rate among different risk groups may also introduce bias.
R: There have also been PCR tests for asymptomatic patients, e.g. for travelers, asymptomatic individuals in health care or other vulnerable sectors, contacts (with criteria of exposure or disposition) etc. This can indeed introduce bias to the assessment of total risks. However, we assume that the assessment of relative risks is unaffected. Therefore, the association between risk factors and the outcome, the estimated odds ratios, should be unbiased.

The authors may consider mentioning the assumptions they made in the manuscript.

*R: Thank you for this valuable suggestion. We have now reformulated our explanation and now write in the methods section: “During the evaluation period from February to the end of September 2020 (i.e., first to third quarter 2020), patients suspected to suffer from COVID-19 infection received naso-pharyngeal swabs for PCR testing in general practice. According to the national testing strategy, participants without symptoms could also be tested in general practice, for example travelers from risk areas, staff in health care or other vulnerable sectors, and contacts of infected persons. However, these cases were to be billed separately by the Ministry and were thus not documented as claims data.” (see lines 75-81). We have also discussed this in the limitations detailed in the discussion section, where we write: “Beyond that, the possibility of asymptomatic participants in a cohort can presumably introduce bias. However, tests of asymptomatic patients were to be billed separately by the government and consequently not documated as claims data for the BASHIP. Therefore, the association between risk factors and the outcome, the estimated odds ratios, should be unbiased in this respect.” (see lines 335-339).*

*In addition, we have corrected one formulation: “… this assumption might be impaired as the cohort of tested participants may also include asymptomatic participants.” (see lines 279-280). We are sorry for the confusing wording before and hope this clears up any confusion.*
